# National trends in age-standardized incidence and mortality rates of acute kidney injury in Peru

**DOI:** 10.1590/2175-8239-JBN-2019-0132

**Published:** 2020-03-23

**Authors:** Percy Herrera-Añazco, Maycol Suker Ccorahua-Ríos, Mirian Condori-Huaraka, Yerika Huamanvilca-Yepez, Elard Amaya, Noé Atamari-Anahui

**Affiliations:** 1 Universidad San Ignacio de Loyola, Unidad de Investigación para la Generación y Síntesis de Evidencias en Salud, Lima, Perú.; 2 Universidad Nacional San Antonio Abad del Cusco, Escuela Profesional de Medicina Humana, Asociación científica de estudiantes de medicina humana (ASOCIEMH CUSCO), Cusco, Perú; 3 Universidad San Ignacio de Loyola, Centro de Excelencia en Investigaciones Económicas y Sociales en Salud, Lima, Perú.; 4 Instituto Nacional de Salud del Niño-Breña, Asociación de Médicos Residentes del Instituto Nacional de Salud del Niño (AMERINSN), Lima, Perú

**Keywords:** Acute Kidney Injury, Epidemiology, Incidence, Mortality, Peru, Lesão Renal Aguda, Epidemiologia, Incidência, Mortalidade, Peru

## Abstract

**Introduction:**

Acute kidney injury (AKI) is a common disorder that causes high healthcare costs. There are limited epidemiological studies of this disorder in low- and middle-income countries. The aim of this study was to describe trends in the age-standardized incidence and mortality rates of AKI in Peru.

**Methods:**

We conducted an ecological study based on a secondary data sources of the basic cause of death from healthcare and death records obtained from establishments of the Ministry of Health of Peru for the period 2005-2016. The age-standardized incidence and mortality rates of AKI were described by region and trend effects were estimated by linear regression models.

**Results:**

During the period 2005-2016, 26,633 cases of AKI were reported nationwide. The age-standardized incidence rate of AKI per 100,000 people increased by 15.2%, from 10.5 (period 2005-2010) to 12.1 (period 2011-2016). During the period 2005-2016, 6,812 deaths due to AKI were reported, which represented 0.49% of all deaths reported for that period in Peru. The age-standardized mortality rate of AKI per 100,000 people decreased by 11.1%, from 2.7 (period 2005-2010) to 2.4 (period 2011-2016). The greatest incidence and mortality rates were observed in the age group older than 60 years.

**Conclusions:**

During the study period, incidence of AKI increased and mortality decreased, with heterogeneous variations among regions.

## RESUMO

**Introdução:**

A lesão renal aguda (LRA) é um distúrbio comum que causa altos custos para a saúde. Existem estudos epidemiológicos limitados sobre esse distúrbio em países de baixa e média renda. O objetivo deste estudo foi descrever as tendências nas taxas de incidência e mortalidade padronizadas por idade da LRA no Peru.

**Métodos:**

Realizamos um estudo ecológico com base em fontes de dados secundárias da causa básica de morte de registros de saúde e óbito obtidos de estabelecimentos do Ministério da Saúde do Peru no período de 2005 a 2016. A incidência padronizada por idade e as taxas de mortalidade por LRA foram descritas por região, e os efeitos de tendência foram estimados por modelos de regressão linear.

**Resultados:**

No período de 2005 a 2016, 26.633 casos de LRA foram relatados em todo o país. A taxa de incidência padronizada de LRA por idade por 100.000 pessoas aumentou 15,2%, de 10,5 (período de 2005 a 2010) para 12,1 (período de 2011 a 2016). Durante o período de 2005 a 2016, foram relatadas 6.812 mortes por LRA, o que representou 0,49% de todas as mortes relatadas para esse período no Peru. A taxa de mortalidade por LRA padronizada por idade por 100.000 pessoas diminuiu 11,1%, de 2,7 (período de 2005 a 2010) para 2,4 (período de 2011 a 2016). As maiores taxas de incidência e mortalidade foram observadas na faixa etária acima de 60 anos.

**Conclusões:**

Durante o período do estudo, a incidência de LRA aumentou e a mortalidade diminuiu, com variações heterogêneas na epidemiologia entre as regiões.

## INTRODUCTION

Acute kidney injury (AKI) is a common and serious clinical condition deriving from several etiologies and associated with high morbidity, mortality, and healthcare costs 1 ^-^ 5. Worldwide, the AKI incidence in adults is 21.6% and the mortality rate is 23.9%, and these indices vary depending on the AKI stage and clinical presentation of the disorder. The incidence is higher in the first stage of AKI and the mortality is higher if the patient requires any renal replacement therapy (RRT). Moreover, the AKI incidence has increased while the AKI mortality has decreased 6. Some studies showed a stabilization of the age-adjusted incidence rate or the incidence among patients requiring hemodialysis, likely related to demographic changes and clinical practice with inpatients 7 ^,^ 8.

Worldwide, it is estimated that 85% of AKI cases are reported in low- and middle-income countries (LMIC); however, more than 80% of epidemiology studies on AKI are conducted in high-income countries. The etiology of AKI varies across countries, likewise AKI mortality is inversely related to healthcare budget and expenditures of countries 2 ^,^ 3 ^,^ 6.

Latin America is one of the most unequal regions worldwide (Gini Index of 52.9, only overcome by Sub-Saharan Africa) and is underrepresented in AKI epidemiology studies 3 ^,^ 6 ^,^ 9. Peru is a Latin American middle-income country. Although its economy and health services coverage have improved, it is still a country with disappointing inequality, with 25% of its population living in poverty, and 6% in extreme poverty 10 ^,^ 11. Although there are some studies in patients with AKI in Peru, these are limited to single-center and patients requiring hemodialysis. 12 ^,^ 13 Likewise, there is no study that assessed the incidence and mortality rates of AKI in Latin American countries.

The objective of our study was to describe trends in incidence and mortality rates of AKI at national and regional level during the period 2005-2016 among patients treated by the Ministry of Health of Peru (MINSA), as a way to contribute to the knowledge on the epidemiology of AKI in middle-income countries.

## METHODS

### STUDY DESIGN

We conducted an ecological study using secondary data sources.

### DATA SOURCES

The data was collected from the national records of cases reported annually: i) cases in healthcare services during period 2005-2016 and ii) deaths based on death certificates during the period 2005-2016 provided by the MINSA. This database contains records of all healthcare interventions conducted within health establishments of MINSA (establishments of the first and second level of care, from regional and national hospitals and specialized institutes), and all deaths occurred in the country recorded by the National Identification Registry of Peru.

Data of the AKI cases was collected from the discharge summary sheets of the hospital and in the health information systems during the outpatient consultation at MINSA facilities nationwide. All cases of AKI and deaths due to AKI recorded with code ICD: N17.0 - N17.9 in MINSA establishments nationwide were included. Cases and deaths that did not have that ICD code were excluded.

### PROCEDURES

We requested to the Platform for Access to Public Information of MINSA the database of reported healthcare interventions by MINSA establishments, as well as deaths records (http://www.minsa.gob.pe/portada/transparencia/solicitud/frmFormulario.asp).

### PARTICIPANTS

The population treated by the MINSA is composed by people who do not have any type of health insurance and those who have comprehensive health insurance, which is around 60% of Peruvian population. Moreover, the MINSA population is characterized by medium and low socioeconomic status, and poverty and extreme poverty conditions 14.

### VARIABLES

The main variables were the incidence and mortality rates of AKI for the period 2005-2016 per 100,000 estimated as: i) cases reported annually in healthcare establishments of MINSA and ii) the number of deaths reported annually. These variables were assessed by year, sex, age group, and region. The population for each region-year were retrieved from the National Institute for Statistics and Informatics of Peru website (https://www.inei.gob.pe/estadisticas/indice-tematico/population-estimates-and-projections/). Likewise, we estimated the MINSA population for each region-year using the National Household Survey of Peru (http://iinei.inei.gob.pe/microdatos/). We also obtained the age-standardized incidence and mortality rates using the direct method based on the population from the World Health Organization for 2000-2025 14.

### DATA ANALYSIS

First, descriptive analysis was done by absolute and relative frequencies of AKI incidence and mortality rates. Second, we conducted an exploratory spatial analysis using the QGIS software v2.10.1 (OSGeo, USA), matching the age-standardized incidence and mortality rates of AKI with geo-referencing of the regions. To this end, we categorized the data in quintiles and averaged the incidence and mortality rates for the first and last six years assessed to reduce the measurement bias associated with one year as reference, following a previous study 15. Finally, we applied linear regression models for each region using the Stata^(®)^ software 15.0 (StataCorp, College Station, USA). The incidence and mortality rates of AKI were the dependent variables and the time was the exposure variable, with the aim of assessing trends for each region. We corrected standard errors by robust variance and considered statistically significant trends with a p<0.05.

### ETHICS STATEMENT

Our study used secondary data sources obtained through a request or public websites. The ethics approval was waived because these data were anonymous, so they did not involve any direct risk of subject identification.

## RESULTS

### TRENDS IN THE INCIDENCE RATE OF AKI

During the period 2005-2016, 26,633 cases of AKI were recorded in the MINSA database (Table 1), of which 13,142 (49.4%) occurred in the age group older than 60 years; 9,162 (34.4%) in the age group of 30 to 59 years, and 4,329 (16.2%) in age group younger than 30 years.

**Table 1:** Absolute and relative frequencies of cases and deaths attributed to AKI recorded in the Ministry of Health of Peru at national level.

Year	Total cases of AKI	Age-standardized incidence rate / 100,000 people	Total number of deaths due to AKI	Frequency of deaths due to AKI*	Age-standardized mortality rate / 100,000 people
2005	1653	9.6	439	0.43	2.3
2006	1700	9.8	532	0.51	2.7
2007	1878	10.9	649	0.61	3.2
2008	2096	11.8	518	0.48	2.5
2009	1980	10.8	588	0.53	2.7
2010	1923	10.1	635	0.59	2.8
2011	1878	9.5	613	0.52	2.7
2012	2761	13.7	677	0.57	2.9
2013	2502	11.9	686	0.55	2.8
2014	2378	10.9	913	0.69	3.6
2015	2781	12.7	232	0.17	0.9
2016	3103	14.0	330	0.23	1.2
Total	26 633	11.3	6812	0.49	2.5

(*) Percentage represented a ratio between the total deaths due to AKI and the total deaths due to all causes in Peru.

**Figure 1:**
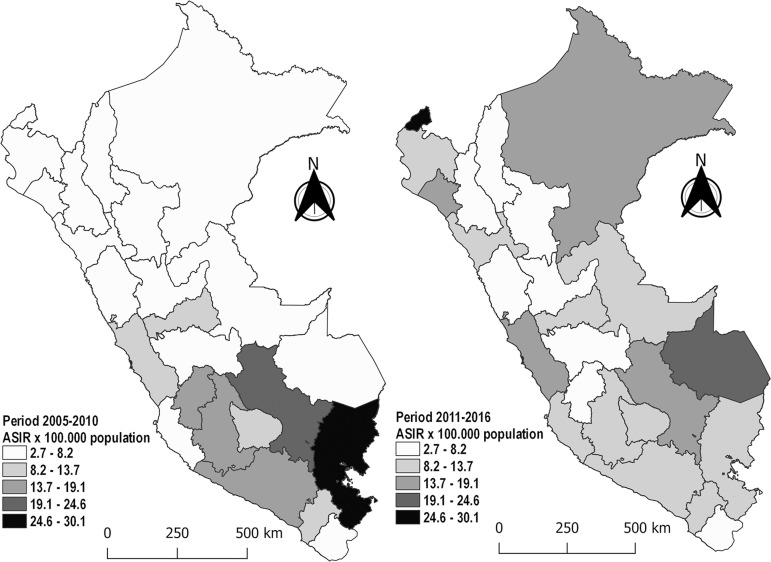
Age-standardized incidence rate (ASIR) of AKI in Peru, by region: comparison between the periods.

The age-standardized incidence rate of AKI in Peru increased from 9.6/100,000 in 2005 to 14.0/100,000 in 2016 (Table 1). The regions with the greatest incidence increase were the Tumbes (542.9%), Loreto (220.6%), and Ucayali (200.2%); while those with the greatest decrease were Huancavelica (-56.9%), Puno (-52.1%), and Huánuco (-36.2%) (Table 2 and Figure 1).

**Table 2:** Age-standardized incidence and mortality rates attributed to AKI recorded in the Ministry of Health of Peru at regional level.

Region	Age-standardized incidence rate / 100,000 people			Age-standardized mortality rate / 100,000 people		
	2005-2010	2011-2016	% change	2005-2010	2011-2016	% change
Peru (country)	10.5	12.1	15.2	2.7	2.4	-11.1
Amazonas	5.3	5.6	6.1	1.3	0.7	-41.8
Ancash	7.4	7.7	4.1	1.9	1.6	-15.6
Apurímac	13.4	10.9	-18.8	4.4	1.6	-62.8
Arequipa	18.0	13.5	-24.8	2.6	3.2	24.3
Ayacucho	13.9	10.5	-24.5	6.1	1.9	-69.3
Cajamarca	5.9	4.5	-23.6	2.0	1.2	-40.3
Callao	30.1	25.9	-13.8	0.8	0.8	-2.2
Cusco	20.8	14.8	-28.8	4.4	2.0	-54.5
Huancavelica	17.6	7.6	-56.9	3.9	2.4	-38.6
Huánuco	6.8	4.3	-36.2	2.6	1.6	-39.4
Ica	5.3	9.6	80.2	0.5	1.3	161.8
Junín	6.1	6.6	7.3	2.2	2.6	20.4
La Libertad	4.5	10.1	122.4	1.0	0.9	-7.3
Lambayeque	5.8	16.2	177.1	1.9	1.4	-23.2
Lima	10.1	16.8	66.3	0.8	0.9	13.5
Loreto	4.8	15.4	220.6	1.3	1.5	11.2
Madre de Dios	7.3	19.6	168.3	0.6	2.1	226.6
Moquegua	11.1	11.0	-1.0	1.6	1.3	-16.4
Pasco	9.5	9.4	-1.0	1.2	1.3	13.1
Piura	3.2	9.1	183.4	0.6	0.5	-3.0
Puno	27.4	13.1	-52.1	11.2	10.1	-10.1
San Martín	4.4	7.2	63.7	1.6	0.9	-41.5
Tacna	5.3	4.9	-7.6	0.7	0.9	21.2
Tumbes	4.4	28.0	542.9	2.0	0.7	-66.5
Ucayali	2.7	8.2	200.2	0.3	0.4	41.9

Linear regression analysis showed higher growing trends of incidence rate in the Tumbes (β=4.58) and Madre de Dios (β=1.99) regions and higher decreasing trends in the Puno (β=-2.19) and Huancavelica (β=-1.48) regions (Table 3).

**Table 3:** Matrix of coefficients by linear regressions: trend effects.

Regions	Incidence of AKI			Mortality of AKI		
	Coeff	95% CI	*p*	Coeff	95% CI	*p*
Amazonas	0.30	[-0.02 , 0.61]	0.064	-0.10	[-0.22 , 0.01]	0.069
Ancash	0.32	[-0.3 , 0.93]	0.279	-0.07	[-0.22 , 0.07]	0.292
Apurimac	-0.29	[-0.81 , 0.23]	0.242	-0.52	[-1.01 , -0.03]	0.039
Arequipa	-0.55	[-2.12 , 1.03]	0.457	0.08	[-0.27 , 0.43]	0.633
Ayacucho	-0.36	[-0.82 , 0.10]	0.114	-0.69	[-0.88 , -0.49]	0.000
Cajamarca	-0.20	[-0.65 , 0.26]	0.360	-0.13	[-0.25 , 0,00]	0.045
Callao	-0.04	[-1.33 , 1.26]	0.953	0.01	[-0.05 , 0.06]	0.806
Cusco	-0.70	[-0.98 , -0.41]	0.000	-0.37	[-0.54 , -0.21]	0.001
Huancavelica	-1.48	[-2.16 , -0.80]	0.001	-0.30	[-0.51 , -0.08]	0.012
Huanuco	-0.33	[-1.53 , 0.87]	0.557	-0.17	[-0.43 , 0.09]	0.175
Ica	0.80	[0.35 , 1.25]	0.003	0.07	[-0.07 , 0.21]	0.279
Junin	0.09	[-0.37 , 0.54]	0.672	-0.03	[-0.25 , 0.20]	0.806
La Libertad	0.97	[0.52 , 1.43]	0.001	-0.02	[-0.05 , 0,00]	0.072
Lambayeque	1.47	[0.71 , 2.22]	0.001	-0.08	[-0.19 , 0.03]	0.129
Lima	0.97	[0.52 , 1.41]	0.001	0.01	[-0.11 , 0.13]	0.821
Loreto	1.41	[0.57 , 2.24]	0.004	0.02	[-0.30 , 0.34]	0.903
Madre de Dios	1.99	[0.97 , 3.01]	0.001	0.18	[0.03 , 0.32]	0.023
Moquegua	0.02	[-0.78 , 0.81]	0.958	0.05	[-0.16 , 0.25]	0.634
Pasco	0.12	[-0.19 , 0.42]	0.405	0.03	[-0.08 , 0.14]	0.584
Piura	0.68	[0.29 , 1.08]	0.003	0.00	[-0.07 , 0.06]	0.942
Puno	-2.19	[-2.90 , -1.48]	0.000	-0.20	[-1.29 , 0.89]	0.697
San Martin	0.53	[0.21 , 0.85]	0.004	-0.10	[-0.22 , 0.02]	0.081
Tacna	0.08	[-0.28 , 0.44]	0.641	0.00	[-0.12 , 0.11]	0.938
Tumbes	4.58	[1.04 , 8.12]	0.016	-0.21	[-0.45 , 0.04]	0.090
Ucayali	1.03	[0.37 , 1.69]	0.006	-0.01	[-0.09 , 0.08]	0.854

Note: All regressions include robust standard errors Coeff: Estimated coefficients CI: Confidence Interval *p*: p value of linear regressions

### TRENDS IN THE MORTALITY RATE OF AKI

Overall, 6,812 deaths due to AKI were reported (Table 1), of which 5,473 (80.3%) occurred in the age group older than 60 years; 961 (14.1%) in the age group of 30 to 59 years, and 378 (5.6%) in age group younger than 30 years.

**Figure 2:**
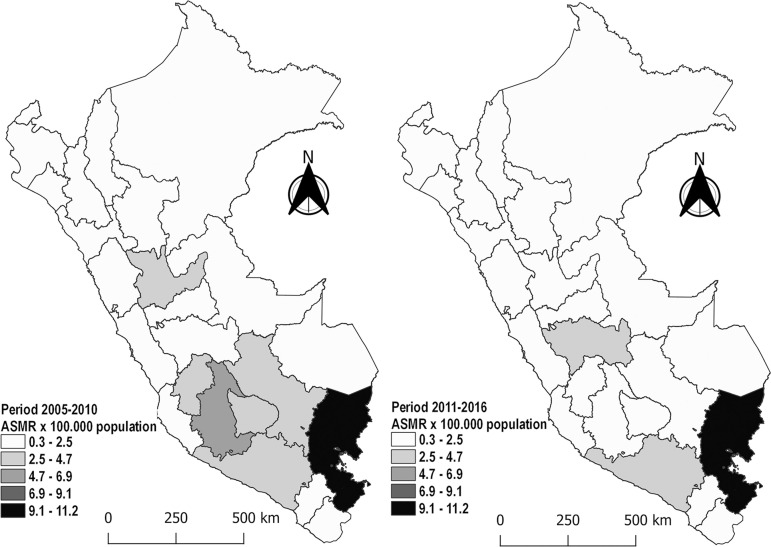
Age-standardized mortality rate (ASMR) of AKI in Peru, by region: comparison between the periods 2005-2010 and 2011-2016.

The age-standardized mortality rate of AKI in Peru decreased from 2.3/100,000 in 2005 to 1.2/100,000 in 2016 (Table 1). The regions with the greatest decrease during the period of analysis were Ayacucho (-69.3%) and Tumbes (-66.5%), while those with the greatest increase were Madre de Dios (226.6%) and Ica (161.8%) (Table 2 and Figure 2).

Linear regression analysis showed increased trend of mortality rate in Madre de Dios (β=0.18) and higher decreasing trends in regions of Ayacucho (β=-0.69) and Apurimac (β=-0.52) (Table 3).

## DISCUSSION

Our study shows an increase in AKI incidence as well as a decrease in AKI mortality. Likewise, the incidence and particulary the mortality were higher in patients older than 60 years.

The increasing trends in AKI incidence nationwide was expected given the increasing incidence of AKI reported in some Latin American countries 3 ^,^ 6; There is no study assessing trends in the incidence of AKI in LMIC, however, it seems that the incidence shows an increasing trend 3.

The incidence reported in our study was significantly lower than the incidence of 3,000 to 5,000 per million population (pmp) reported in high-income countries, but similar to 102 pmp in 33 studies conducted in Latin America as reported in the 0by25 Initiative of the International Society of Nephrology 3. Although they state that at least one study from Peru was included, this is not described. In general, they highlighted that critical patients were overrepresented 3. This may explain the difference with our study, since the national sample we used did not discriminate between critical and non-critical patients, and worldwide, significantly differences are reported between cases of community-acquired AKI and AKI in intensive care units (8.3% and 31.7%, respectively) 6.

In addition, it is possible that in a healthcare system with infrastructure problems and shortage of nephrologists for early diagnosis 16 ^-^ 17, the reported cases are concentred in stage 3 of AKI that needed RRT and not early stage of AKI (2.3% compared to 16.3%). 6. The patients requiring RRT are elders 18, which could explain the greatest incidence of AKI in our study among patients older than 60 years. These patients are likely younger than the 2.3% of patients with AKI requiring RRT reported worldwide, since the proportion of these patients is lower in LMIC than high-income countries 3.

Regions with higher incidence increase were those in the tropical areas (Tables 2 and 3, and Figure 1). This could be due to the risk of illnesses such as severe gastroenteritis and endemic infections complications such as malaria, leptospirosis and dengue 2 ^,^ 19 ^,^ 20, which are common in these regions. Problems related to environmental sanitation, such as contaminated water, are also common, which would increase the risk of AKI 3 ^,^ 19 ^,^ 20.

On the other hand, the decline trends of mortality associated with AKI in our study is similar to that reported worldwide; however, the profile of mortality in LMIC has particular characteristics 6. In LMIC the mortality rate is lower than in high-income countries because patients are younger, have less comorbid diseases, and AKI derives in general from one etiology -as in our results-, and it is likely that cases are more severe when the patient is older and requires some kind of RRT 2 ^,^ 3 ^,^ 19 ^,^ 20. Although there are reports of an increased mortality in critical patients with AKI requiring hemodialysis in high-income countries 19, the profile of comorbid conditions, ethnicity, and etiology of AKI in these countries could be different compared to LMIC, making mortality rate constant or lower 2 ^,^ 3 ^,^ 19 ^,^ 20. On the other hand, although the coverage of hemodialysis for AKI in Latin America has improved 21, in our country, there is still poor coverage 22, and it is possibly underreported in many regions.

As with the incidence, there was a heterogeneous decrease in mortality among regions, which could be associated with a shortage of healthcare staff, limited access to healthcare services, and limitations in the diagnosis and treatment options 9 ^,^ 17, especially because nephrologists and treatment centers are concentrated in Lima 17 ^,^ 22. Madre de Dios reported the greatest mortality rate due to AKI (Tables 2 and 3, and Figure 2), this could be related to the harmful effects of illegal mining activities in this region 23 ^,^ 24.

Our study has several limitations. First, we used secondary data sources, which could have underreported data; however, during the last years there has been an improvement in the quality of records and information systems in Peru 25 ^,^ 26. Second, we only used coding for AKI diagnosis, which has a low sensitivity to quantify the disease burden, apart from not evaluating other clinical variables such as etiology, comorbid conditions, or severity 27. However, several studies on AKI epidemiology included more than 50% of patients with a definition of AKI based on codifications 6. Third, no patient from private health establishments or the social security system were included, which could underestimate the incidence of AKI. Despite these limitations, the strength of our study is that it reports national and regional trends of AKI epidemiology, and the results may be used as a preliminary study for further studies in Latin America to address other aspects related to this illness 9.

## CONCLUSION

During the period 2005-2016, the age-standardized incidence rate of AKI increased, especially in the Tumbes, Loreto, Ucayali, and Madre de Dios regions. Moreover, there was a heterogeneous decline in mortality, which was significantly higher in the Ayacucho, Tumbes, and Apurimac regions. Finally, the greatest proportion of cases and deaths were recorded among patients older than 60 years.
